# Supplementary material for: Architecture and regulation of filamentous human cystathionine beta-synthase
Source: Nat Commun. 2024 Apr 4;15:2931. doi: 10.1038/s41467-024-46864-x (PMC10995199; doi:10.1038/s41467-024-46864-x)
Supplement: Supplementary file 1 — Supplementary Information [file 41467_2024_46864_MOESM1_ESM.pdf]

Supplementary Information

Architecture and regulation of filamentous human cystathionine beta-synthase

Thomas J. McCorvie[1,5]*, Douglas Adamoski[2], Raquel A. C. Machado[2], Jiazhi Tang[3], Henry J. Bailey[1,6], Douglas S. M. Ferreira[1,3], Claire Strain-Damerell[1,7], Arnaud Baslé[3], Andre L. B. Ambrosio[4], Sandra M. G. Dias[2], Wyatt W. Yue[1,5]*

[1]Centre for Medicines Discovery, Nuffield Department of Clinical Medicine, University of Oxford, Oxford OX3 7DQ, UK
[2]Brazilian Biosciences National Laboratory, Brazilian Center for Research in Energy and Materials, 13083-970, Campinas, Brazil
[3]Biosciences Institute, The Medical School, Newcastle University, Newcastle upon Tyne, NE2 4HH, UK
[4]Sao Carlos Institute of Physics, University of Sao Paulo, Sao Carlos, SP, Brazil
[5]Present address: Biosciences Institute, The Medical School, Newcastle University, Newcastle upon Tyne, NE2 4HH, UK
[6]Present address: Institute of Biochemistry II, Faculty of Medicine, Goethe University Frankfurt, Frankfurt, Germany
[7]Present address: Research Complex at Harwell, Harwell Science and Innovation Campus, Didcot, OX11 0FA, UK

*Correspondence e-mail: thomas.mccorvie@newcastle.ac.uk; wyatt.yue@newcastle.ac.uk

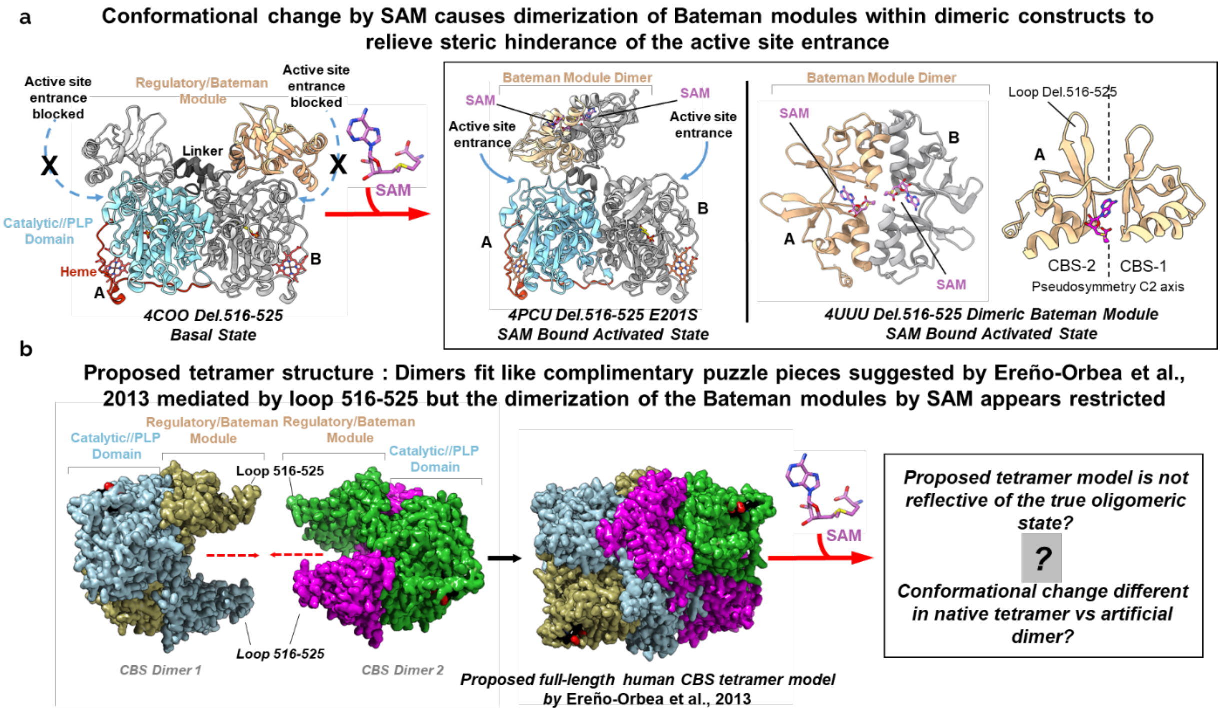

Supplementary Fig. 1 | Previous crystal structures of CBS$^{\Delta 516–525}$ without and with SAM, and the proposed tetrameric state. a, Crystal structures of dimeric CBS$^{\Delta 516–525}$ without and with SAM demonstrating a domain un-swapping and dimerization of the Bateman modules in the SAM bound activated state. In the basal state CBS is a domain swapped dimer where the neighboring subunits regulatory/Bateman module sits atop the catalytic site entrance. Two crystal structures, one of a mutant and another of the loop-deleted regulatory domain alone, show that SAM binding causes disassociation of the regulatory domain from the catalytic domain resulting in dimerization to form a Bateman module dimer. Note that in the 4PCU crystal structure the Bateman module dimer is tilted relative to the catalytic dimer due to crystal packing and both active sites have increased accessibility. SAM is only found in the S2 site, a binding pocket between the CBS motifs on one face of the regulatory domain. The putative S1 site on the opposite face is unoccupied. **b**, Ereño-Orbea et al., 2013 suggested that CBS oligomerizes by clasping interactions of loop 516-525 from two domain swapped dimers to form an intimate tetrameric particle. However, this proposed architecture is not compatible with the known crystal structures of the SAM bound activated state. This suggests that the proposed tetrameric state is incorrect, or the known conformational change is different within the context of the full-length CBS tetramer.

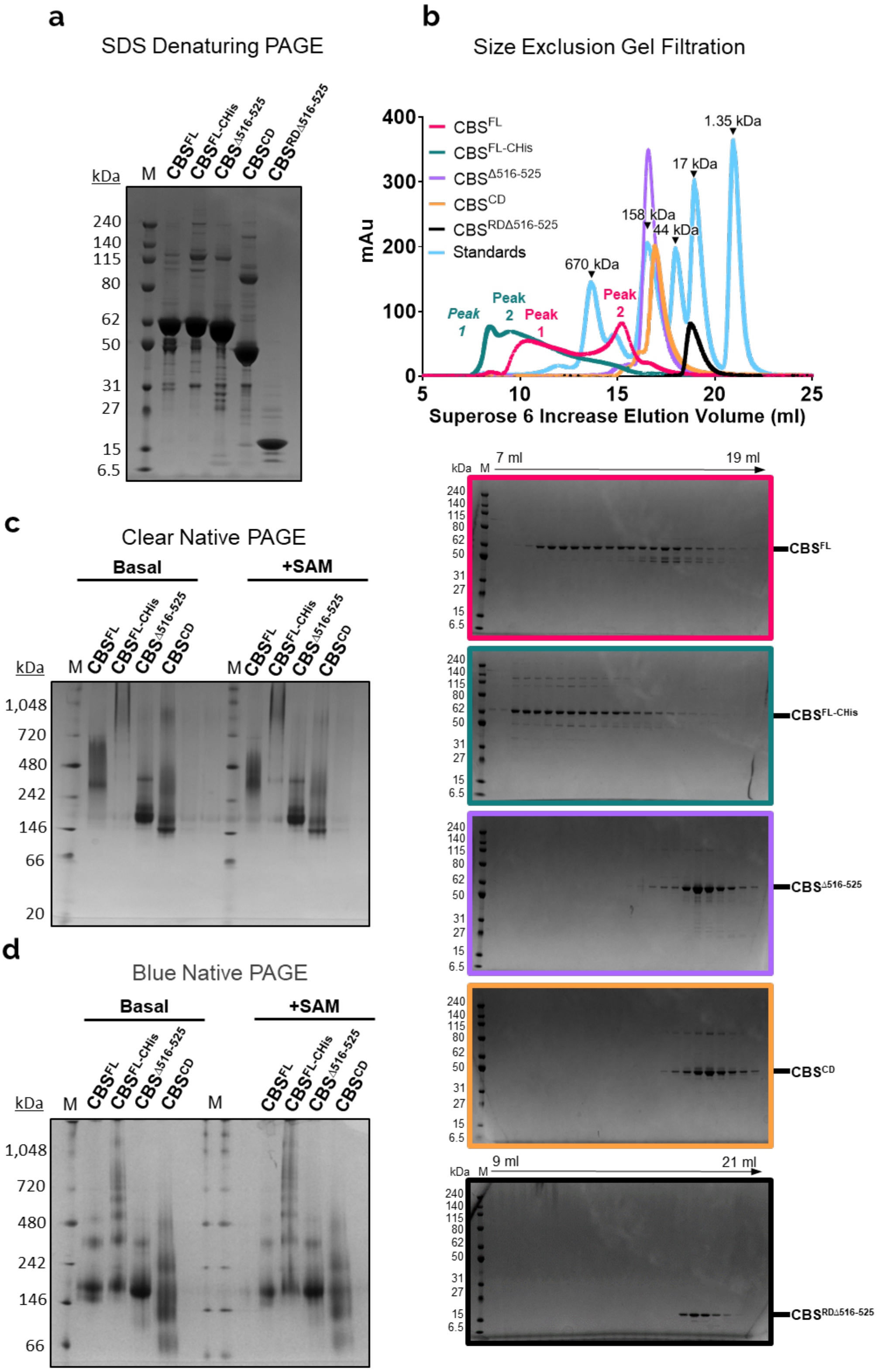

Molecular Weight Standards Equation

$LogMW = -5.715 * K_{av} + 8.164$

e

	K_{av}	LogMW	Approx. Molecular Weight	Subunit Predicted Molecular Weight	Number of Subunits	Apparent Oligomeric State
**CBS^{FL} Peak 1**	0.160407	7.24694	17.7 mDa	60 kDa	295	Oligomer
**CBS^{FL} Peak 2**	0.468491	5.486178	306.2 kDa	60 kDa	5.1	Tetramer
**CBS$^{FL\text{-}CHis}$ Peak 1**	0.040102	7.93451	86.0 mDa	61 kDa	1409.8	Oligomer
**CBS$^{FL\text{-}CHis}$ Peak 2**	0.104392	7.567078	36.9 mDa	61 kDa	604.9	Oligomer
**CBS$^{\Delta 516\text{-}525}$**	0.555697	4.98778	97.2 kDa	59 kDa	1.6	Dimer
**CBS^{CD}**	0.579885	4.849539	70.7 kDa	45 kDa	1.5	Dimer
**CBS$^{RD\Delta 516\text{-}525}$**	0.695735	4.187434	15.4 kDa	17 kDa	0.905882353	Monomer

Supplementary Fig. 2 | Analysis of the oligomeric states of different CBS constructs. a, Coomassie stained SDS-PAGE of the five CBS constructs used in this study. **b**, Superose 6 gel filtration chromatograph and Coomassie stained SDS-PAGE gels of each CBS constructs. n = 1. **c**, Representative clear-native PAGE of each CBS construct without and with 1 mM SAM. n = 2 technical repeats. **d,** Representative blue-native PAGE of each CBS construct without and with 1 mM SAM. n = 2 technical repeats. **e,** Table of calculated molecular weight and number of subunits of each CBS construct from analytical gel filtration. These values were calculated using the known molecular weights of the standards and their elution volumes as described in the methods. Source data for Supplementary Fig. 2a-2d are provided as a Source Data File.

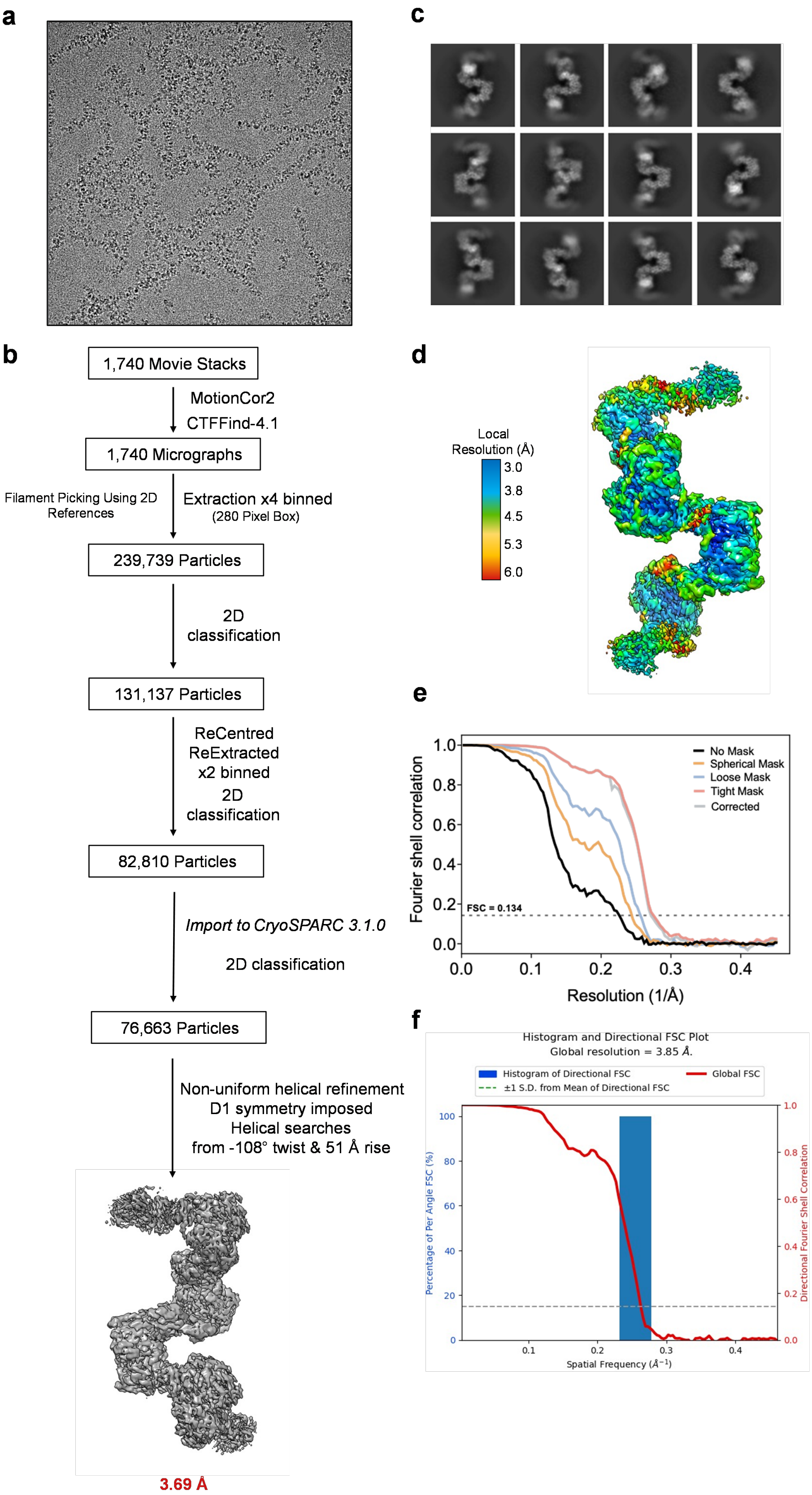

Supplementary Fig. 3 | Helical cryo-EM data processing of basal state CBS$^{FL\text{-}CHis}$. a, Representative Falcon 3 micrograph of CBS$^{FL\text{-}CHis}$. **b**, Processing flow chart of CBS$^{FL\text{-}CHis}$ in the basal state. **c**, Representative helical 2D classes of CBS$^{FL\text{-}CHis}$. **d**, Local resolution variation of the 3.7 Å helical CBS$^{FL\text{-}CHis}$ basal state map. **e**, Fourier shell correlation (FSC) curve. **f**, Directional FSC plot of the helical CBS$^{FL\text{-}CHis}$ map.

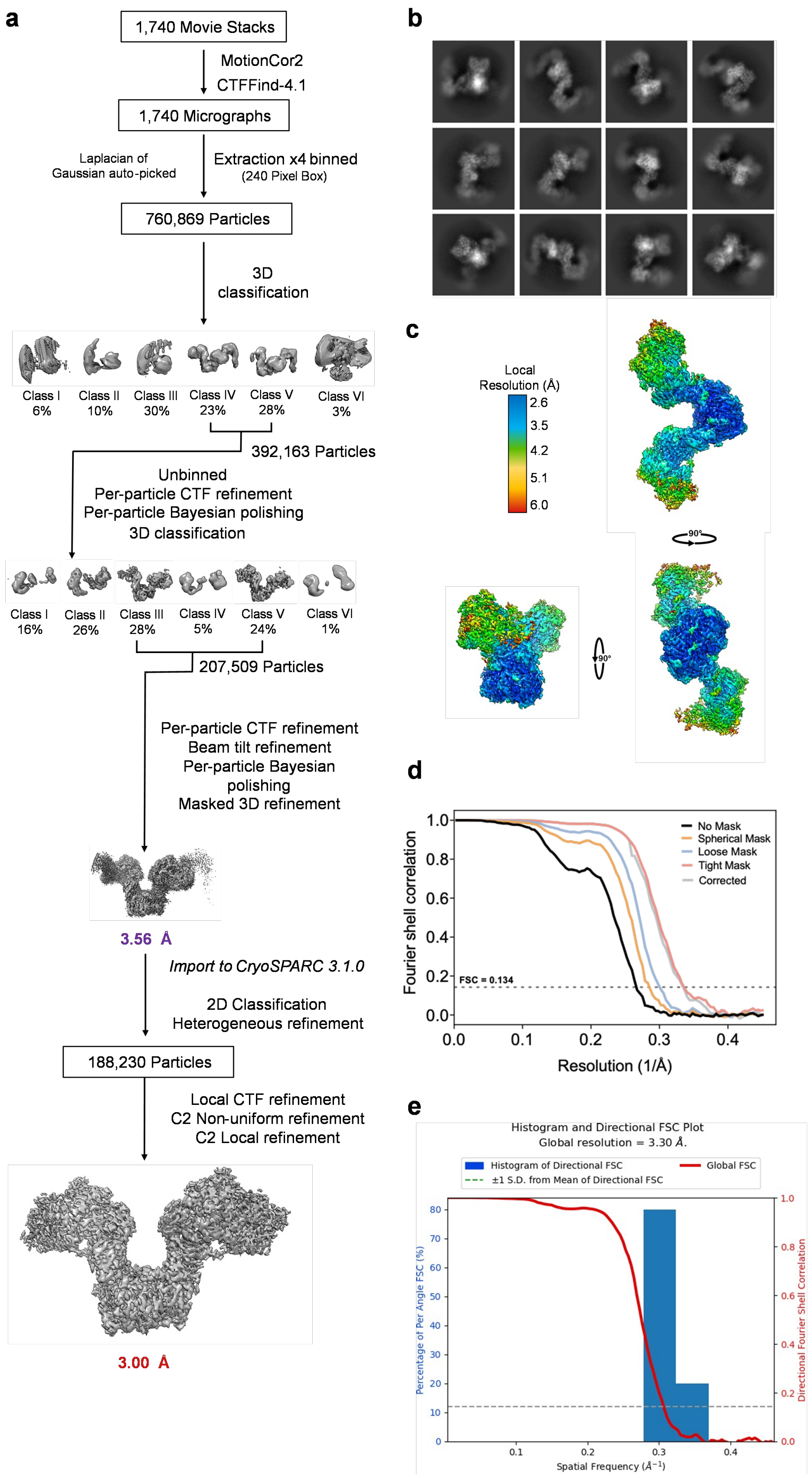

Supplementary Fig. 4 | Single particle cryo-EM data processing of basal state $CBS^{FL-CHis}$. **a**, Processing flow chart of $CBS^{FL-CHis}$ in the basal state. **b**, Representative 2D classes of $CBS^{FL-CHis}$. **c**, Local resolution variation of the 3.0 Å $CBS^{FL-CHis}$ basal state map. **d**, Fourier shell correlation (FSC) curve. **e**, Directional FSC plot of the 3.0 Å $CBS^{FL-CHis}$ map.

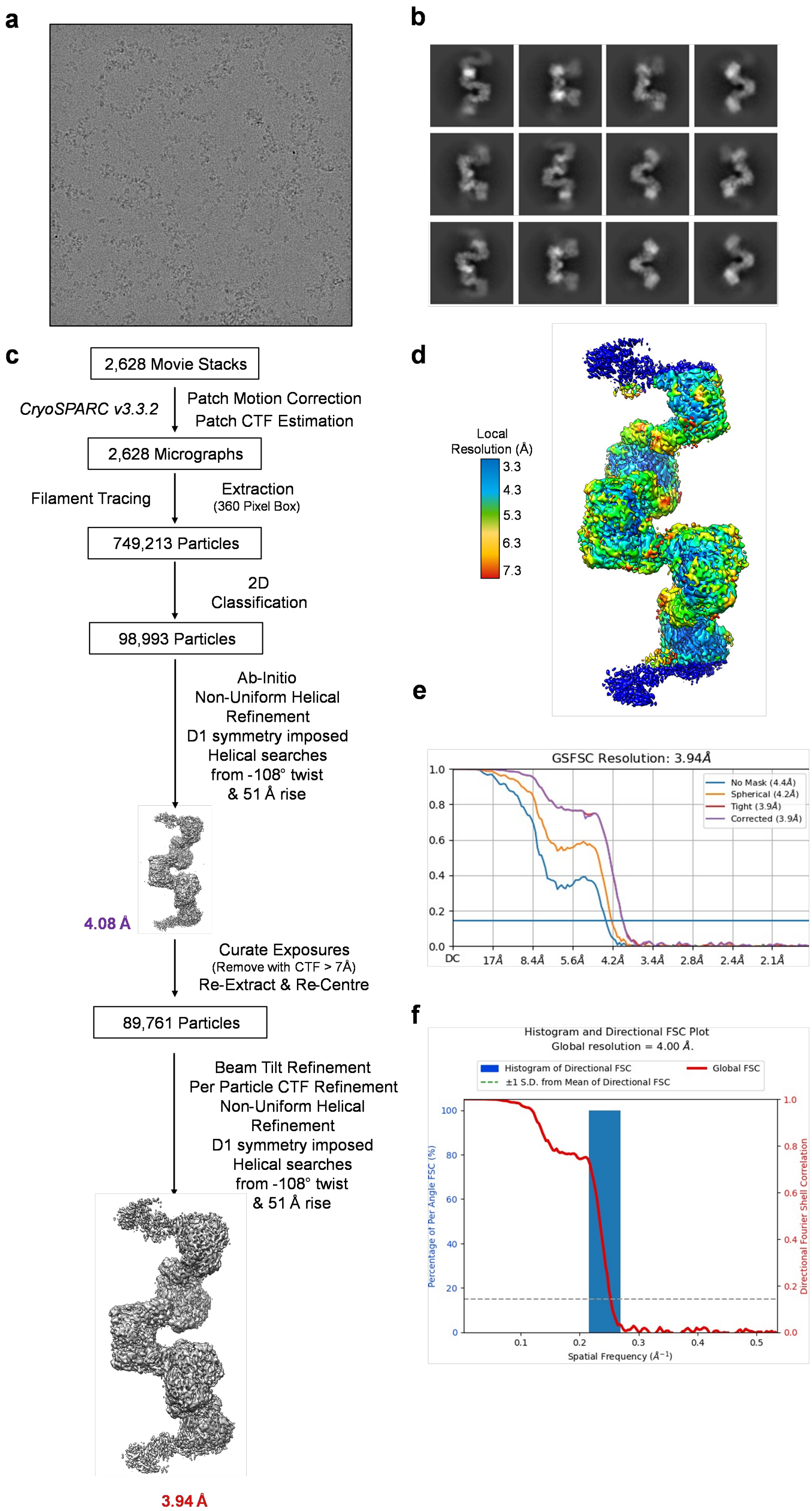

Supplementary Fig. 5 | Helical cryo-EM data processing of basal state CBS^{FL}. a, Representative Falcon 4 micrograph of CBS^{FL}. **b**, Processing flow chart of CBS^{FL} in the basal state. **c**, Representative helical 2D classes of CBS^{FL}. **d**, Local resolution variation of the 3.9 Å helical CBS^{FL} basal state map. **e**, Fourier shell correlation (FSC) curve. **f**, Directional FSC plot of the 3.9 Å helical CBS^{FL} map.

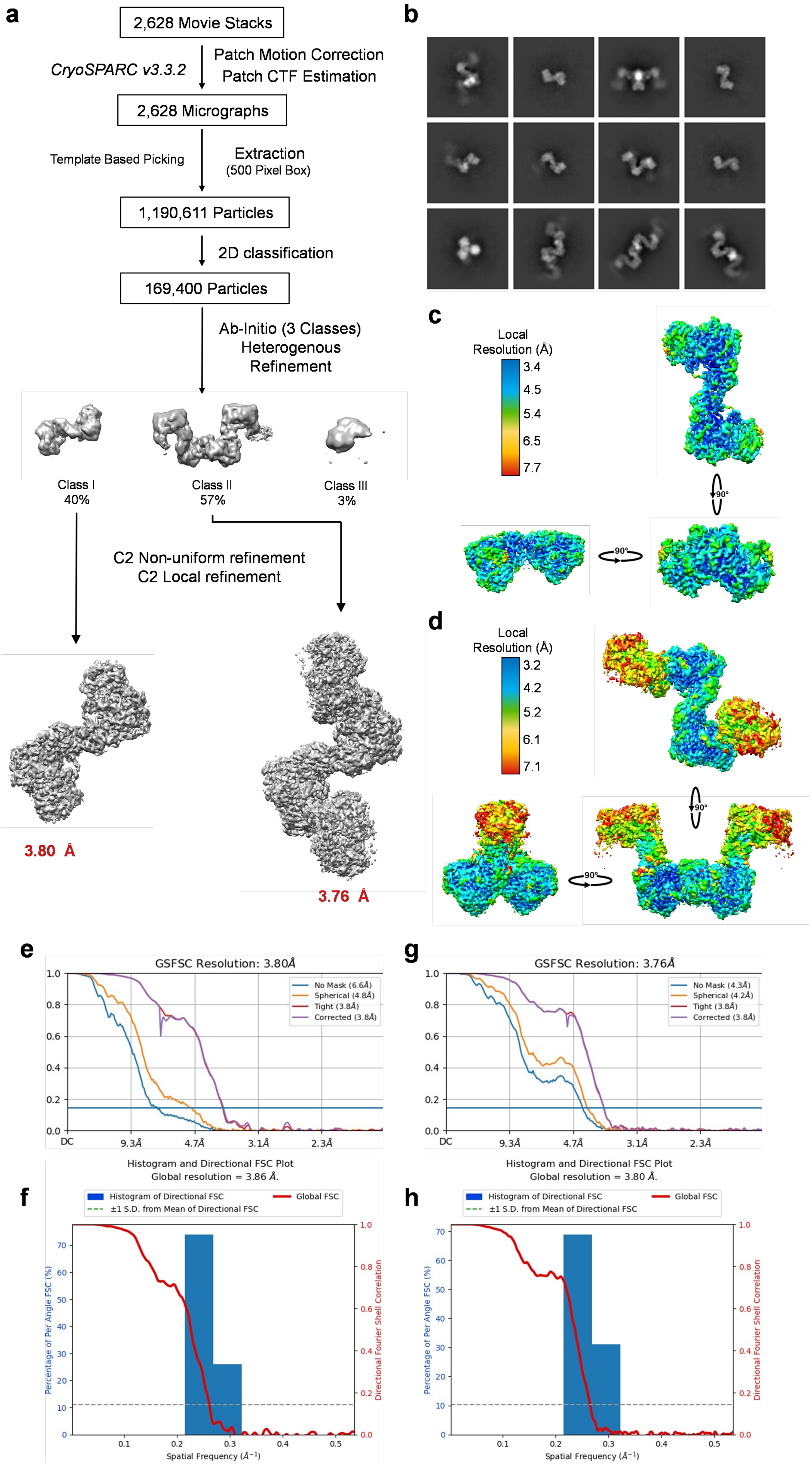

Supplementary Fig. 6 | Single particle cryo-EM data processing of basal state CBS^{FL}. a, Processing flow chart of CBS^{FL} in the basal state. **b**, Representative 2D classes of CBS^{FL}. **c, d,** Local resolution variation of the two CBS^{FL} basal state maps. **e, f,** Fourier shell correlation (FSC) curves. **f, h,** Directional FSC plots of the two CBS^{FL} maps.

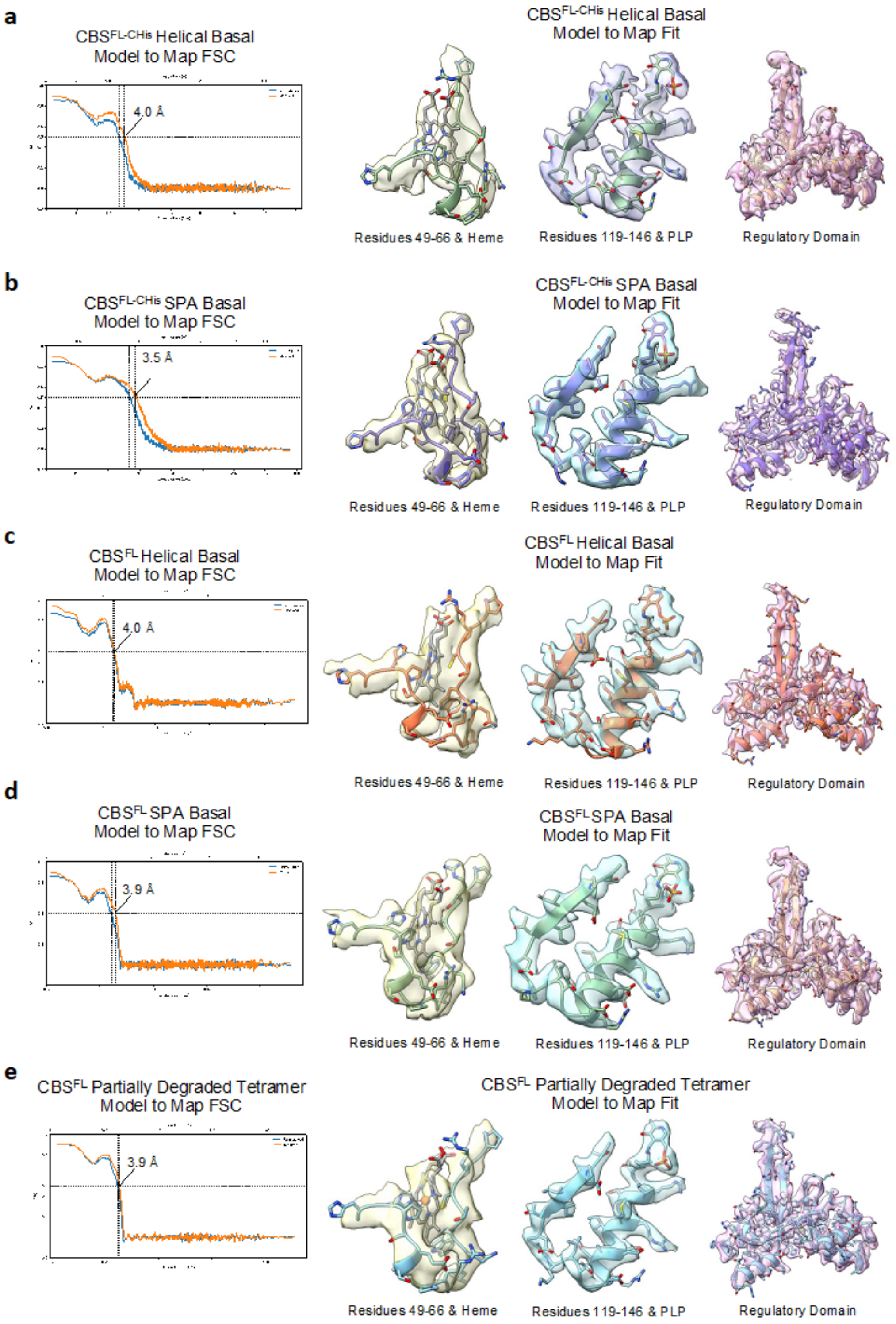

Supplementary Fig. 7 | Modelling of the basal state of CBS. a, Model to map FSC curve and examples of model fit into the EM density for the CBS$^{FL\text{-}CHis}$ helical basal state. **b**, Model to map FSC curve and examples of model fit into the EM density for the CBS$^{FL\text{-}CHis}$ SPA basal state. **c**, Model to map FSC curve and examples of model fit into the EM density for the CBS^{FL} helical basal state. **d**, Model to map FSC curve and examples of model fit into the EM density for the CBS^{FL} SPA basal state. **e**, Model to map FSC curve and examples of model fit into the EM density for the CBS$^{FL\text{-}CHis}$ partially degraded tetramer basal state.

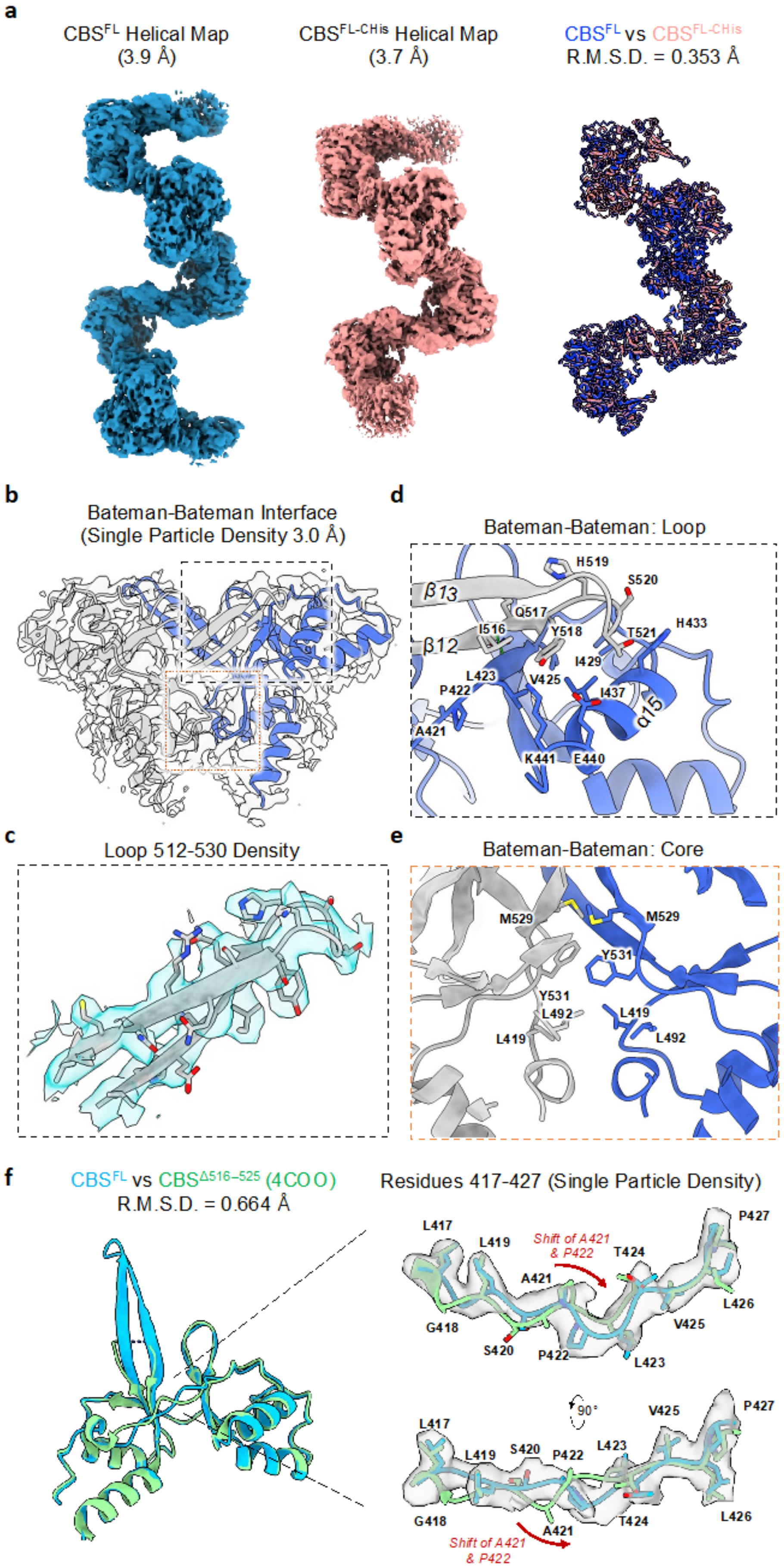

a
CBS^FL Helical Map (3.9 Å)
CBS^FL-CHis Helical Map (3.7 Å)
CBS^FL vs CBS^FL-CHis
R.M.S.D. = 0.353 Å
b
Bateman-Bateman Interface
(Single Particle Density 3.0 Å)
c
Loop 512-530 Density
d
Bateman-Bateman: Loop
β13
β12
H519
S520
Q517
I516
Y518
T521
H433
I429
L423
V425
P422
I437
α15
A421
K441
E440
e
Bateman-Bateman: Core
M529
M529
Y531
Y531
L492
L419
L419
L492
f
CBS^FL vs CBS^Δ516–525 (4COO)
R.M.S.D. = 0.664 Å
Residues 417-427 (Single Particle Density)
L417
L419
Shift of A421 & P422
T424
P427
A421
L426
G418
V425
S420
P422
L423
90°
V425
P427
L417
L419
S420
P422
L423
G418
A421
T424
L426
Shift of A421 & P422

Supplementary Fig. 8 | Key residues are involved in the polymerization of human CBS. **a**, Cryo-EM maps of both CBS^{FL} and $CBS^{FL\text{-}CHis}$ determined using helical reconstruction. Alignment of the resulting refined atomic models show very little difference. **b**, Density and model fit at the Bateman-Bateman interface of the 3.0 Å resolution single particle map of $CBS^{FL\text{-}CHis}$. **c**, Close up of the cryo-EM density and residues of the loop 512-530 at the Bateman-Bateman interface. **d**, Close up of residues involved in interactions at the loop 516-525 of the Bateman-Bateman interface. **e**, Close up of residues involved in hydrophobic interactions at the core of the Bateman-Bateman interface. **f**, Structural alignment of the regulatory domains from the cryo-EM map of filamentous $CBS^{FL\text{-}CHis}$ and crystal structure of dimeric $CBS^{\Delta 516\text{–}525}$ (4COO). Interactions due to filamentation result in a shift of the residues 417-423.

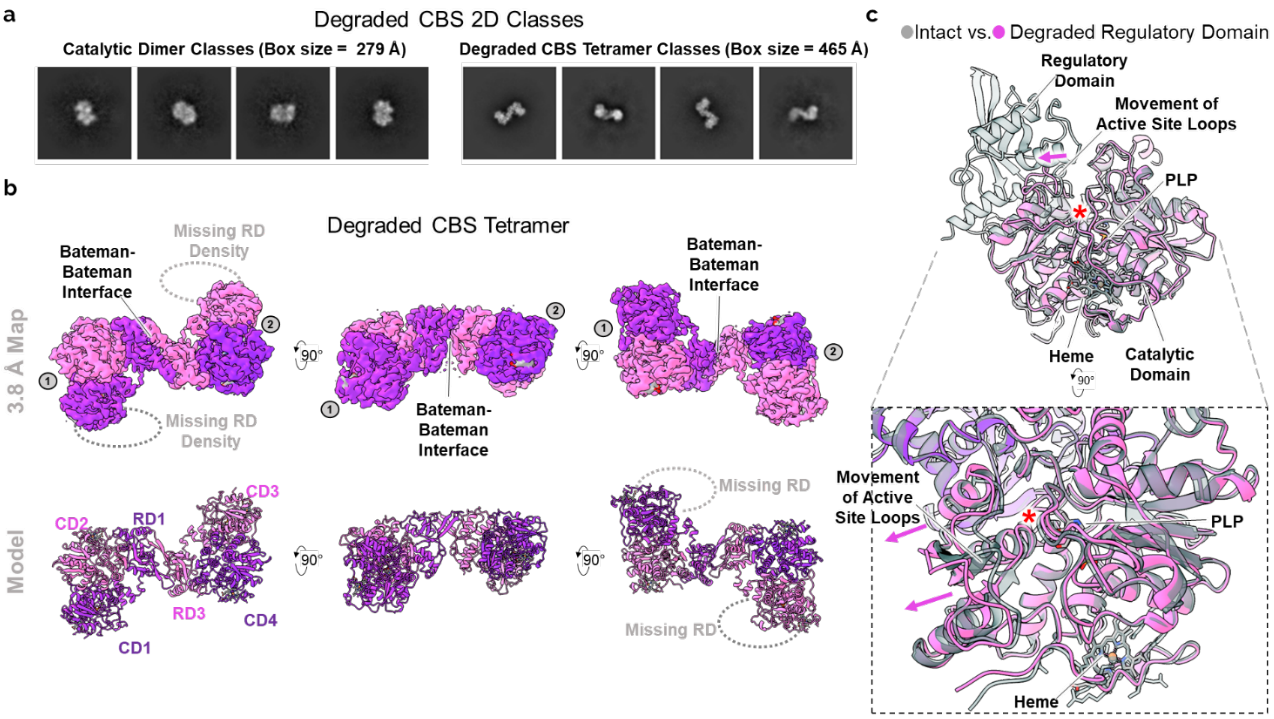

Supplementary Fig. 9 | CBS^{FL} degrades into a tetramer and dimer. a, Example 2D classes representative of degraded CBS catalytic domain dimer and degraded tetrameric CBS. **b**, Multiple views of the 3.8 Å resolution map and model of tetrameric degraded CBS. The tetramer is formed by Bateman-Bateman interactions of two CBS heterodimers consisting of one full-length protomer (residues 42-548) and one degraded protomer without the regulatory domain (residues 42-398). The regulatory domain has been proteolyzed at the flexible linker (residues 382-411) between it and the catalytic domain. **c**, Structural alignment of intact CBS^{FL} vs degraded CBS. The absence of the regulatory domain allows the active site loops to open and increases accessibility to the active site denoted as the red asterisk.

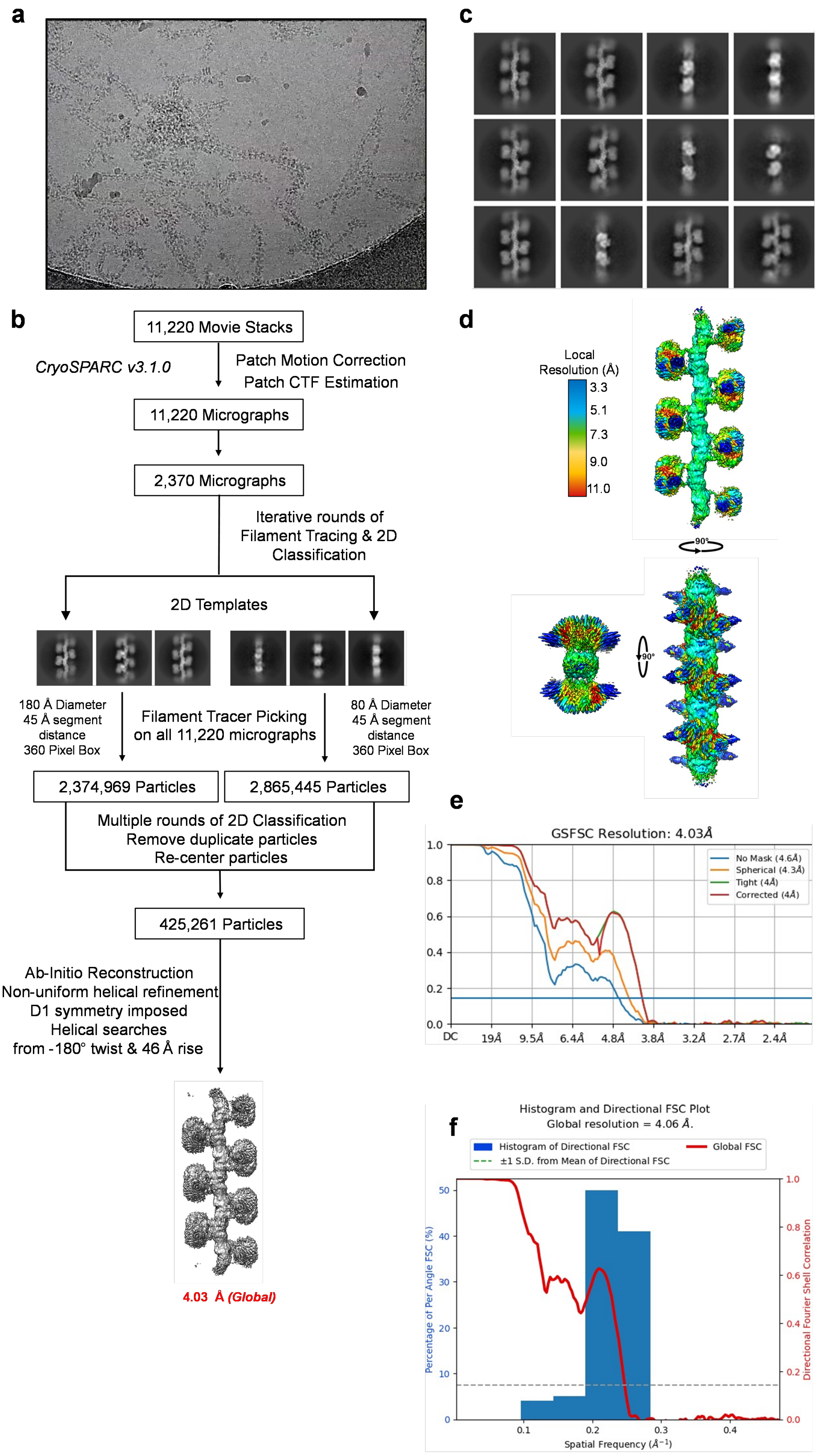

Supplementary Fig. 10 | Helical cryo-EM data processing of activated state CBS$^{FL\text{-}CHis}$. **a**, Representative K3 micrograph of CBS$^{FL\text{-}CHis}$ in the presence of SAM. **b**, Processing flow chart of CBS$^{FL\text{-}CHis}$ in the activated, SAM bound, state. **c**, Representative helical 2D classes of CBS$^{FL\text{-}CHis}$ bound to SAM. **d**, Local resolution variation of the 4.0 Å helical CBS$^{FL\text{-}CHis}$ activated state map. **e**, Fourier shell correlation (FSC) curve. **f**, Directional FSC plot of the helical CBS$^{FL\text{-}CHis}$ plus SAM map.

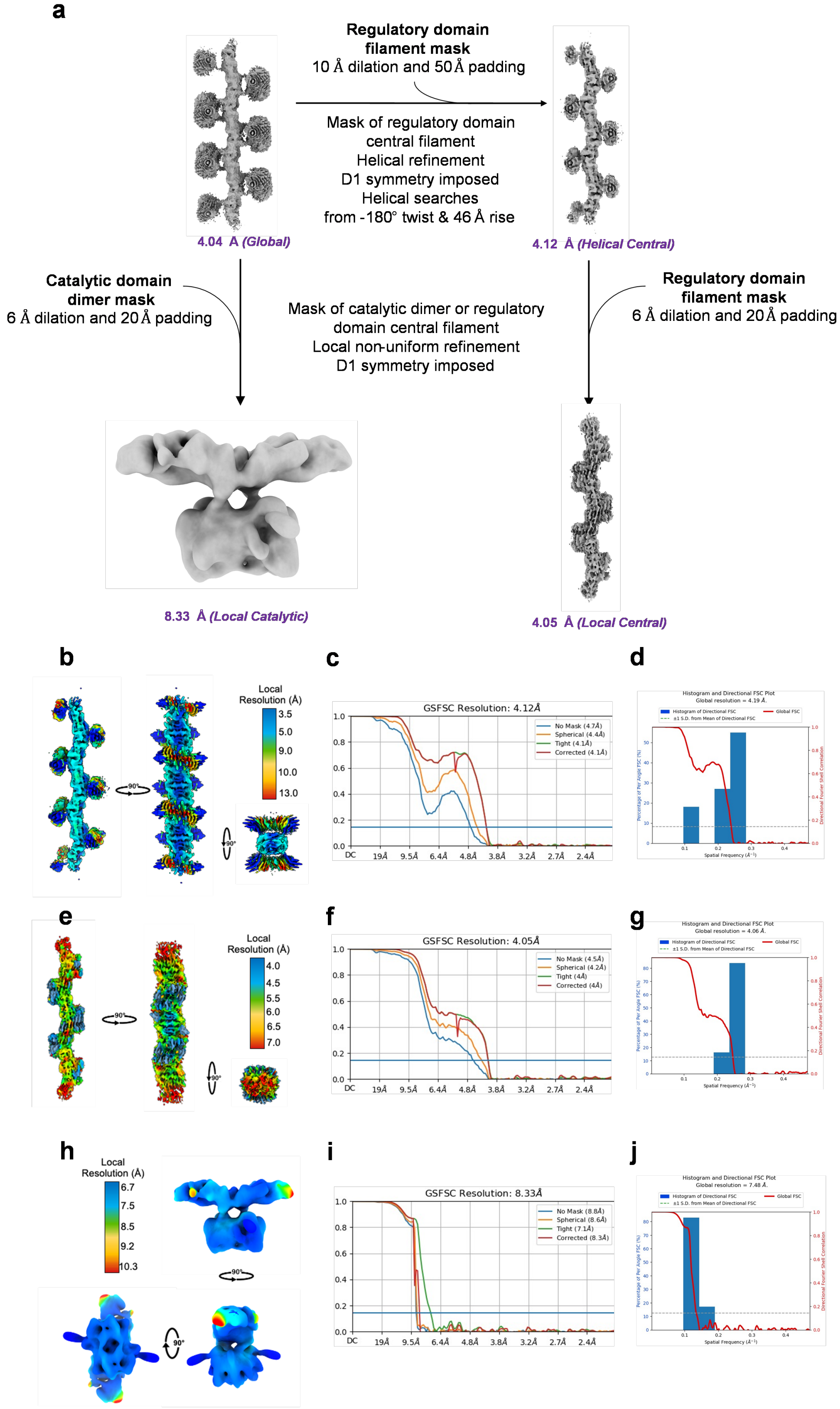

Supplementary Fig. 11 | Further cryo-EM data processing of activated state CBS$^{FL\text{-}CHis}$. **a**, Local helical and single particle processing flow chart of CBS$^{FL\text{-}CHis}$ in the activated, SAM bound, state. **b-d**, Local resolution, FSC curve, and directional FSC plot of the helical central map of CBS$^{FL\text{-}CHis}$ plus SAM. **e-g**, Local resolution, FSC curve, and directional FSC plot of the local central map of CBS$^{FL\text{-}CHis}$ plus SAM. **h-j**, Local resolution, FSC curve, and directional FSC plot of the local catalytic map of CBS$^{FL\text{-}CHis}$ plus SAM.

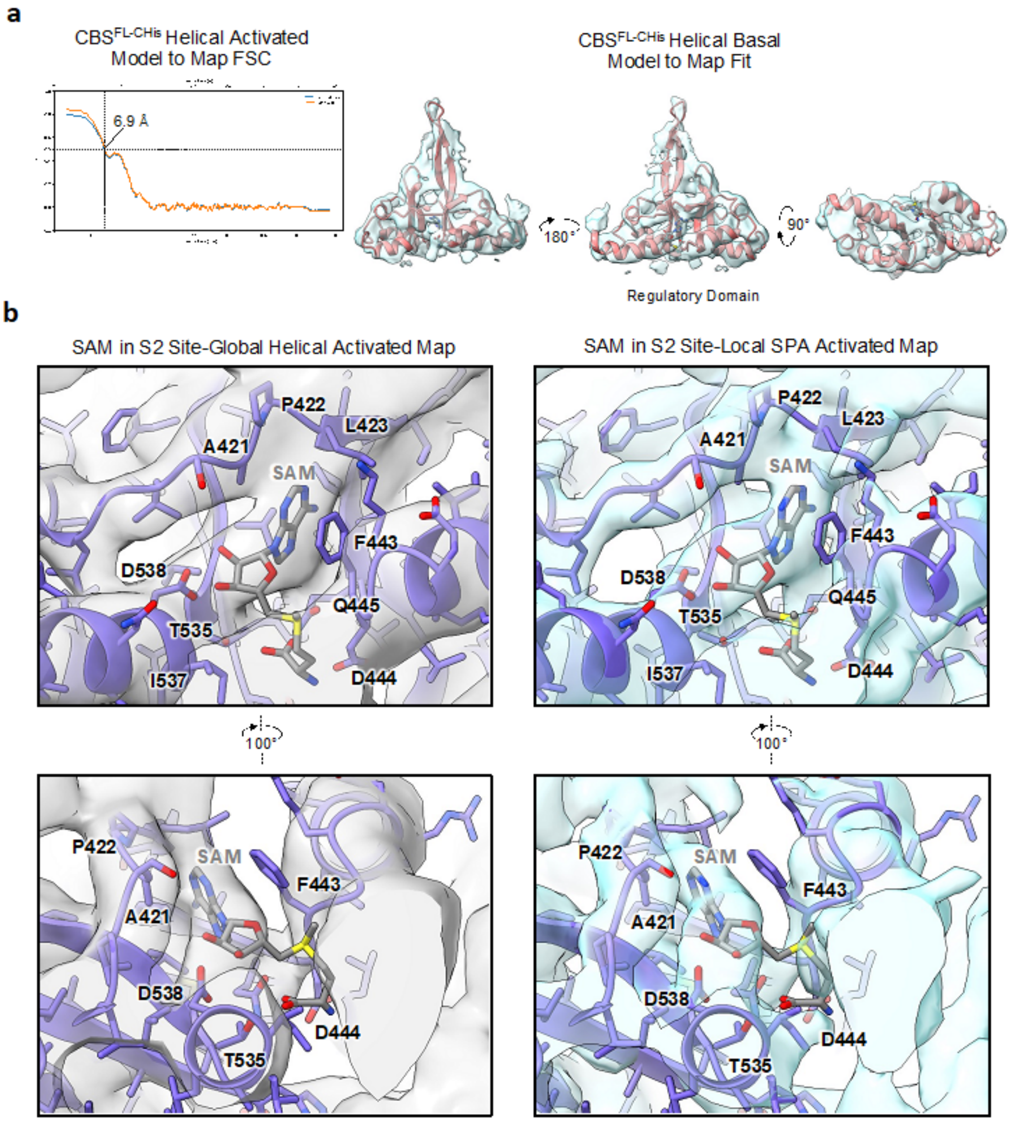

Supplementary Fig. 12 | Modelling of activated SAM bound state of CBS$^{FL\text{-}CHis}$. a, Model to map FSC curve against the global helical map. Model to map fit is also shown for one regulatory domain chain in the global helical map showing the presence of secondary structure elements. **b,** The SAM bound in the S2 site and the surrounding residues. Density is shown both for the global helical and local SPA refinement activated maps.

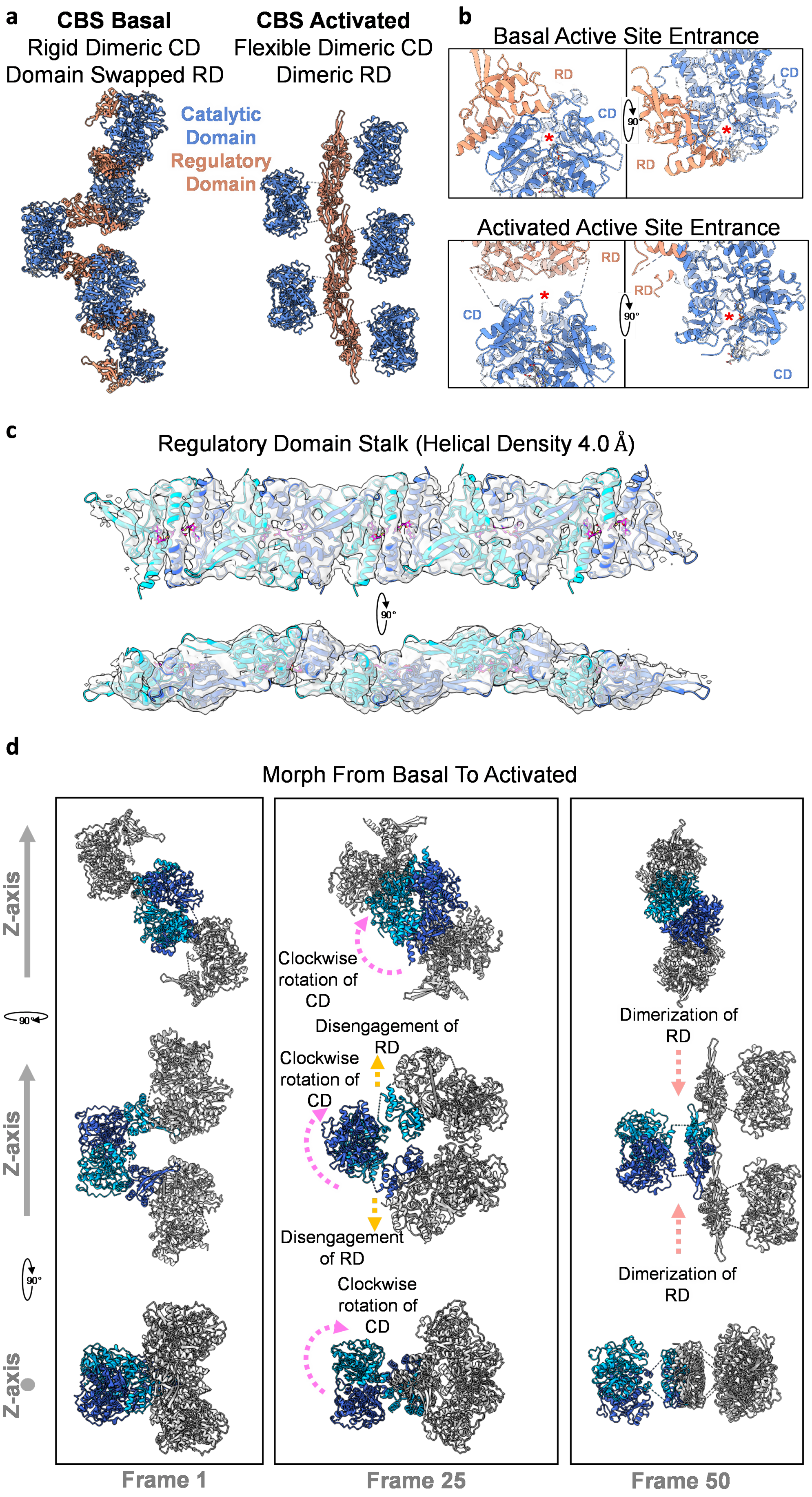
a
CBS Basal
Rigid Dimeric CD
Domain Swapped RD
CBS Activated
Flexible Dimeric CD
Dimeric RD
Catalytic Domain
Regulatory Domain
b
Basal Active Site Entrance
RD
CD
90°
Activated Active Site Entrance
c
Regulatory Domain Stalk (Helical Density 4.0 Å)
d
Morph From Basal To Activated
Z-axis
Clockwise rotation of CD
Disengagement of RD
Dimerization of RD
Frame 1
Frame 25
Frame 50

Supplementary Fig. 13 | The allosteric change initiated by SAM binding is compatible with the filamentous architecture of human CBS. a, Structures of the basal and SAM bound activated state showing the relative positions of the catalytic and regulatory domains. **b**, Close-up of the active site entrance in both basal and activated states. The active site entrance is denoted as a red asterisk. **c**, Cryo-EM density, and model fit of the central regulatory domain stalk of the activated state. **d**, A morph of one turn of the CBS filament from the basal to activated state. Both models when aligned to the central Z-axis suggest that the conformational change due to SAM requires rotation of the catalytic domain along with disengagement and dimerization of the regulatory domains.

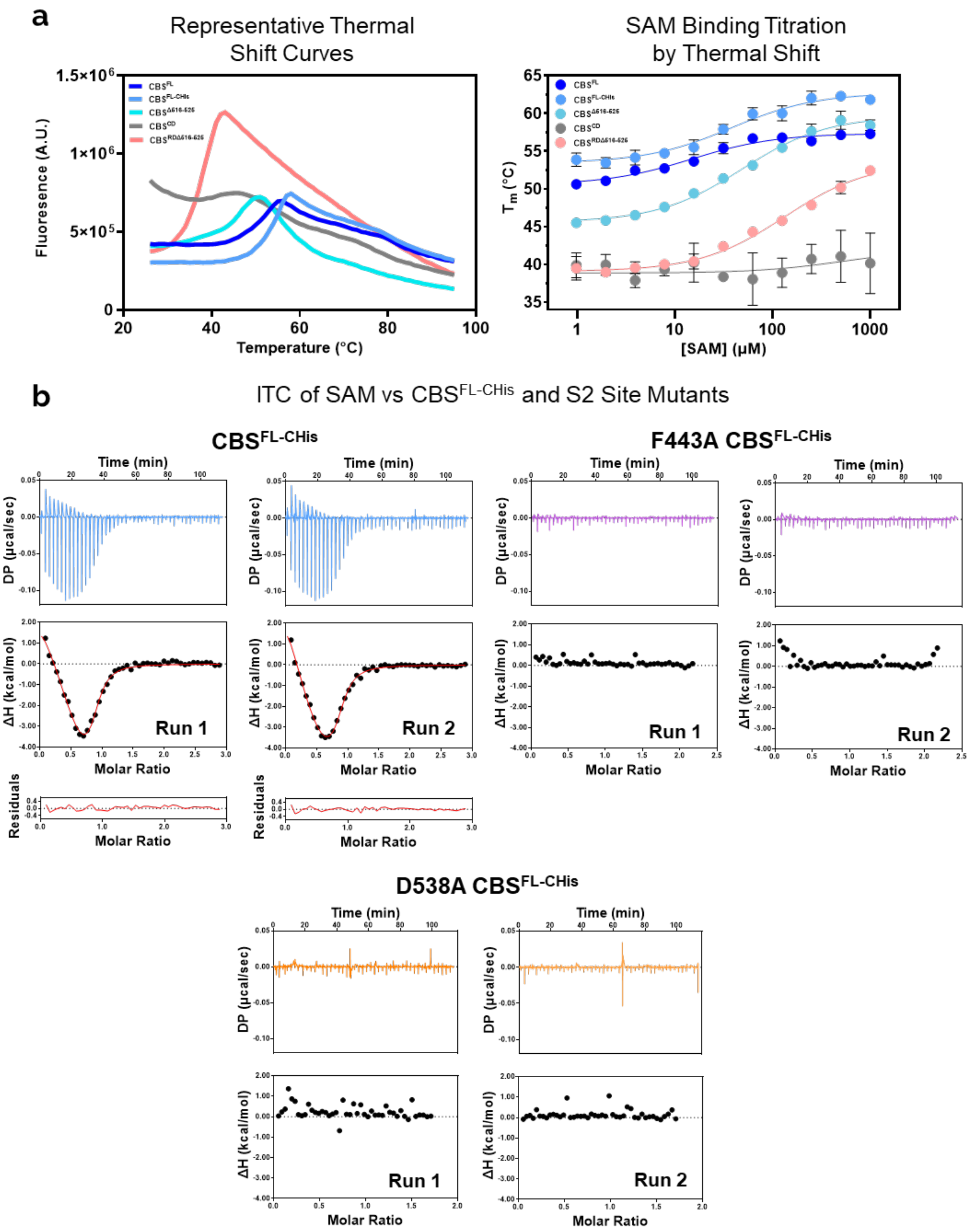

Supplementary Fig. 14 |Thermal shift of CBS constructs along with ITC analysis of SAM titrations into CBS$^{FLC\text{-}His}$ and S2 site mutants. a, Representative thermal shift curves and SAM titrations for CBS$^{FL\text{-}CHis}$, CBS^{FL}, CBS$^{\Delta 516\text{–}525}$, CBS^{CD}, and CBS$^{RD\Delta 516\text{–}525}$ **b**, Replicate ITC titrations of SAM against wild-type, F443A, and D538A CBS$^{FL\text{-}CHis}$. Binding parameters were determined by fitting to two site binding. Red line represents fit of experimental data (dots) into the two-site binding model. Plots of residuals of fit are shown when appropriate. Run 1 is

presented in Figure 3 and is shown here for comparison purposes only. Source data for Supplementary Fig. 14a and 14b are provided as a Source Data File.

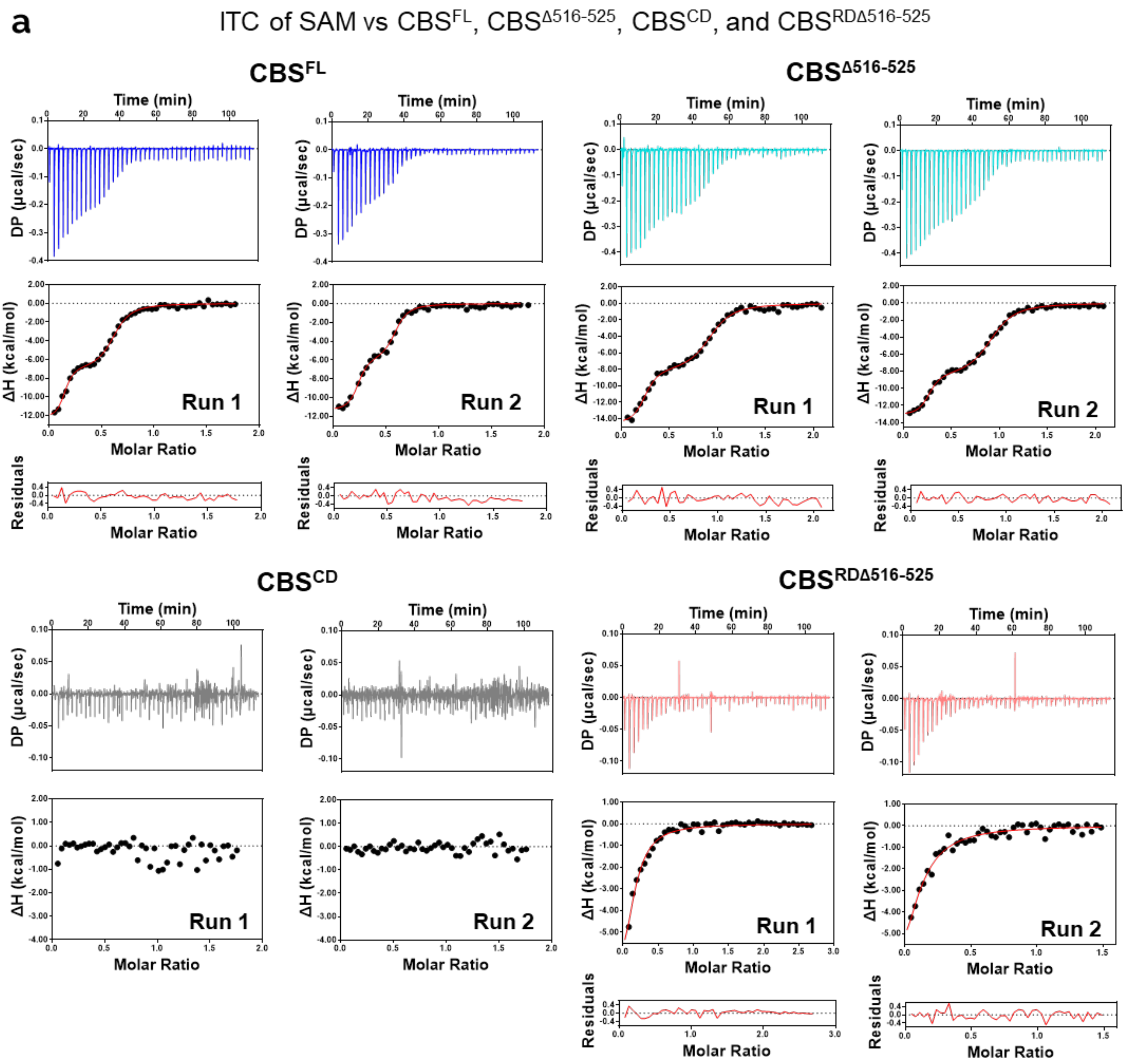

b

Construct	First Event/Site n[1]	K_d[1] (nM)	ΔH[1] (kcal/mol)	-TΔS[1] (kcal/mol)	Second Event/Site n[2]	K_d[2] (nM)	ΔH[2] (kcal/mol)	-TΔS[2] (kcal/mol)
**CBS$^{FL\text{-}CHis}$**	0.34 ± 0.07	161 ± 1	4.8 ± 0.9	-14.1 ± 0.9	0.54 ± 0.06	644 ± 14	-6.7 ± 0.6	-1.75 ± 0.6
**CBS^{FL}**	0.23 ± 0.01	16.2 ± 1.4	-12.0 ± 0.78	1.34 ± 0.71	0.41 ± 0.06	668 ± 96	-6.1 ± 1.0	-1.9 ± 1.2
**CBS$^{Δ516\text{-}525}$**	0.24 ± 0.01	24.9 ± 2.9	-14.0 ± 1.13	3.61 ± 1.18	0.70 ± 0.02	1030 ± 101	-8.1 ± 0.2	-0.1 ± 0.3
**CBS$^{RDΔ516\text{-}525}$**	0.14 ± 0.02	4015 ± 120	-10.4 ± 0.08	2.67 ± 0.06	N.A.	N.A.	N.A.	N.A.

Supplementary Fig. 15 | ITC analysis of SAM titrations into constructs representative of the domain arrangement of CBS. a, Replicate ITC titrations of SAM against CBS^{FL}, CBS$^{Δ516–525}$, CBS^{CD}, and CBS$^{RDΔ516–525}$. Red line represents fit of experimental data (dots) into the one or two-site binding model. Plots of residuals of fit are shown when appropriate. **b**, Calculated ITC parameters for SAM binding against CBS$^{FL\text{-}CHis}$, CBS^{FL}, CBS$^{Δ516–525}$, and CBS$^{RDΔ516–525}$. Binding parameters were determined by fitting to one or two site binding

appropriately. Values are means and ± s.d. of $n = 2$ technical repeats. Source data for Supplementary Fig. 15a are provided as a Source Data File.

a

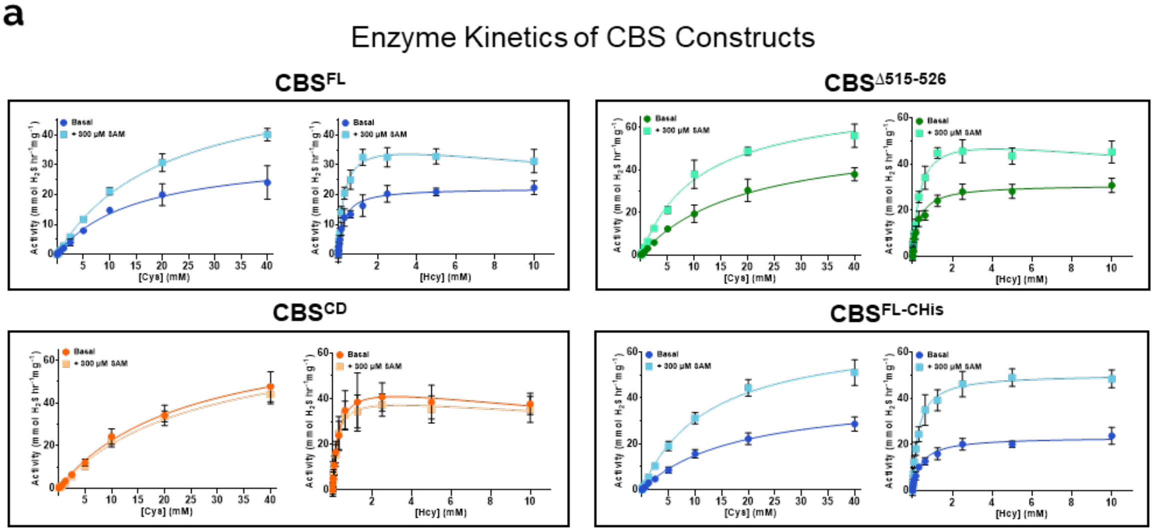

		Basal		+300 μM SAM	
		Cysteine (Cys)	Homocysteine (Hcy)	Cysteine (Cys)	Homocysteine (Hcy)
**CBS^{FL}**	V_{max} (mmol H_2S hr^{-1} mg^{-1})	**33.9 ± 1.9**	**22.1 ± 0.5**	**61.2 ± 1.7**	**39.1 ± 1.6**
	K_m (mM)	**15.1 ± 1.9**	**0.29 ± 0.03**	**20.4 ± 1.1**	**0.30 ± 0.03**
	K_i (mM)	**N.A.**	**N.A.**	**N.A.**	**41.5 ± 11.3**
**CBS$^{Δ516-525}$**	V_{max} (mmol H_2S hr^{-1} mg^{-1})	**57.1 ± 2.9**	**31.0 ± 0.7**	**74.0 ± 2.5**	**54.5 ± 2.3**
	K_m (mM)	**19.3 ± 2.0**	**0.33 ± 0.03**	**10.8 ± 0.9**	**0.36 ± 0.04**
	K_i (mM)	**N.A.**	**N.A.**	**N.A.**	**47.5 ± 16.4**
**CBS^{CD}**	V_{max} (mmol H_2S hr^{-1} mg^{-1})	**76.6 ± 4.04**	**48.15 ± 3.2**	**73.7 ± 3.0**	**41.9 ± 2.4**
	K_m (mM)	**24.2 ± 2.5**	**0.28 ± 0.5**	**25.6 ± 2.0**	**0.20 ± 0.04**
	K_i (mM)	**N.A.**	**35.7 ± 16.6**	**N.A.**	**52.6 ± 29.7**
**CBS$^{FL-CHis}$**	V_{max} (mmol H_2S hr^{-1} mg^{-1})	**41.8 ± 1.6**	**23.2 ± 0.6**	**69.0 ± 1.8**	**50.4 ± 0.9**
	K_m (mM)	**17.9 ± 1.5**	**0.44 ± 0.05**	**12.6 ± 0.8**	**0.29 ± 0.02**
	K_i (mM)	**N.A.**	**N.A.**	**N.A.**	**N.A.**

b

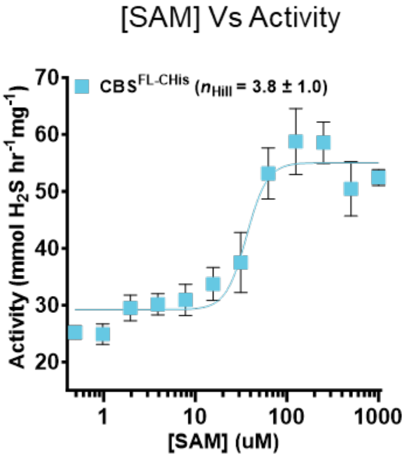

	Minimum Activity (mmol H_2S hr^{-1} mg^{-1})	Maximum Activity (mmol H_2S hr^{-1} mg^{-1})	n_{Hill}	K_{act} (μM)
**CBS^{FL}**	**22.5 ± 0.5**	**40.0 ± 0.5**	**3.0 ± 0.6**	**26.0 ± 2.1**
**CBS$^{Δ516-525}$**	**37.1 ± 0.9**	**55.0 ± 1.0**	**2.0 ± 0.6**	**29.3 ± 4.9**
**CBS^{CD}**	**N.A.**	**N.A.**	**N.A.**	**N.A.**
**CBS$^{FL-CHis}$**	**29.2 ± 0.7**	**55.0 ± 0.9**	**3.8 ± 1.0**	**36.2 ± 2.6**

Supplementary Fig. 16 | The enzyme activity and SAM activation of the CBS constructs in this study. a, Enzyme kinetics of CBS^{FL}, CBS$^{Δ516–525}$, CBS^{CD}, and CBS$^{FL-CHis}$ without (Basal) and with SAM (+ 300 μM SAM). Mean values and error bars are ± s.d. of n = at least 6 technical repeats. **b**, H_2S producing activity of CBS$^{FL-CHis}$ in response to increasing amounts of SAM. Values are means and error bars are ± s.d. of n = at least 4 technical repeats **c**, Fitted values for the allosteric activation by SAM of CBS^{FL}, CBS$^{Δ516–525}$, CBS^{CD}, and CBS$^{FL-CHis}$. Values are means and ± s.d. of n = at least 4 technical repeats. Source data for Supplementary Fig. 16 are provided as a Source Data File.

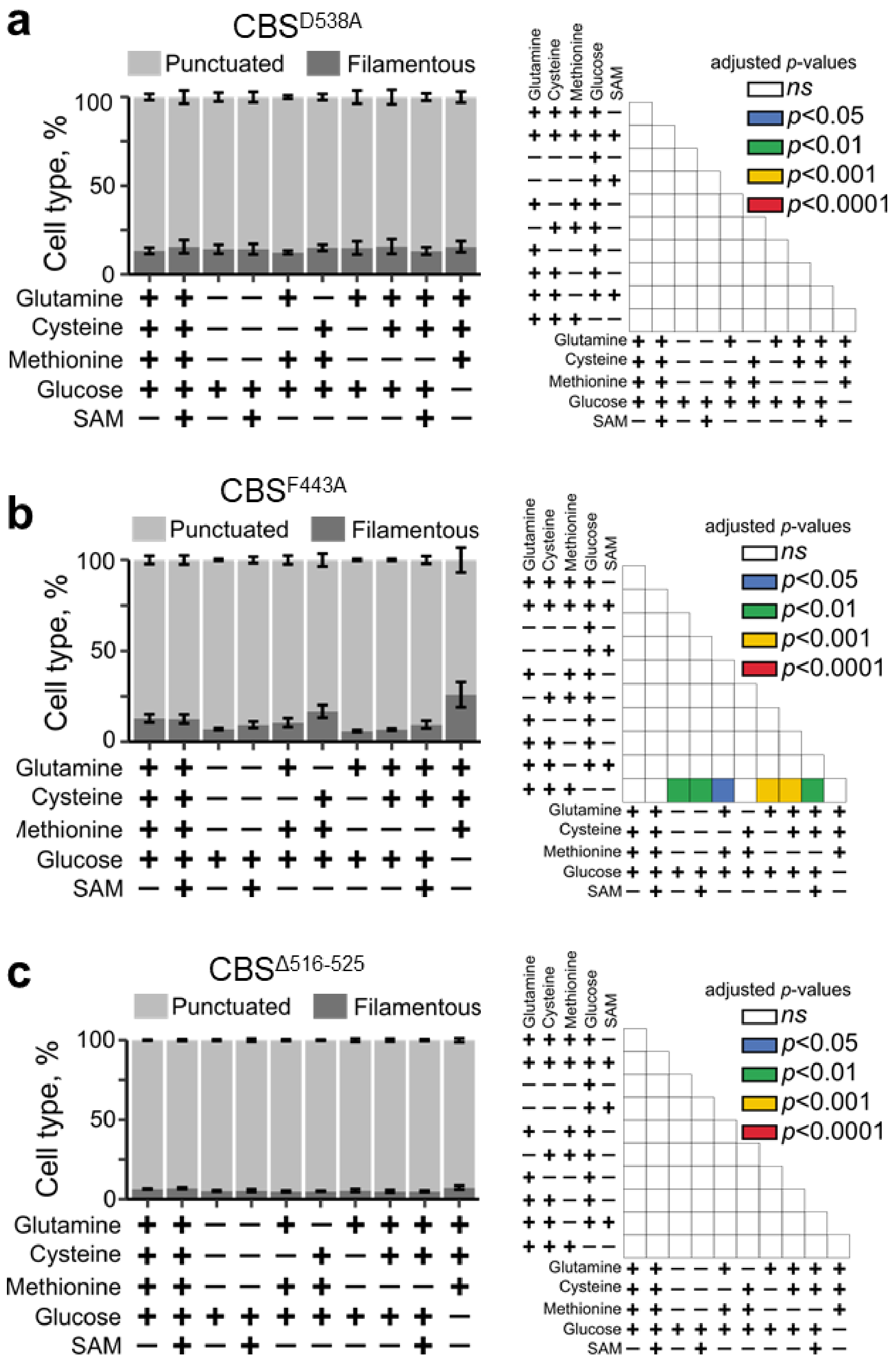

Supplementary Fig. 17 | The Effect of SAM Binding Impairment on CBS Filamentation in PC-3 Cells. PC-3 cells transfected with mKO2-CBS^{D538A} (a), mKO2-CBS^{F443A} (b), and mKO2-CBS$^{Δ516-525}$ (c), incubated in either complete or nutrient-depleted medium for 8 hours. Cells were categorized by their predominant morphology - filamentous or punctuated, determined using fractal-D complexity analysis. Quantifications from n = 6 wells; ≥65 cells (a), ≥158 cells (b), and ≥459 cells (b) per replicate. Mean and s.e.m. are shown; statistical significance is shown via one-way ANOVA and Tukey's test. Significance for on the right for all plots. NS = not significant. Source data for Supplementary Fig. 17 are provided as a Source Data File.

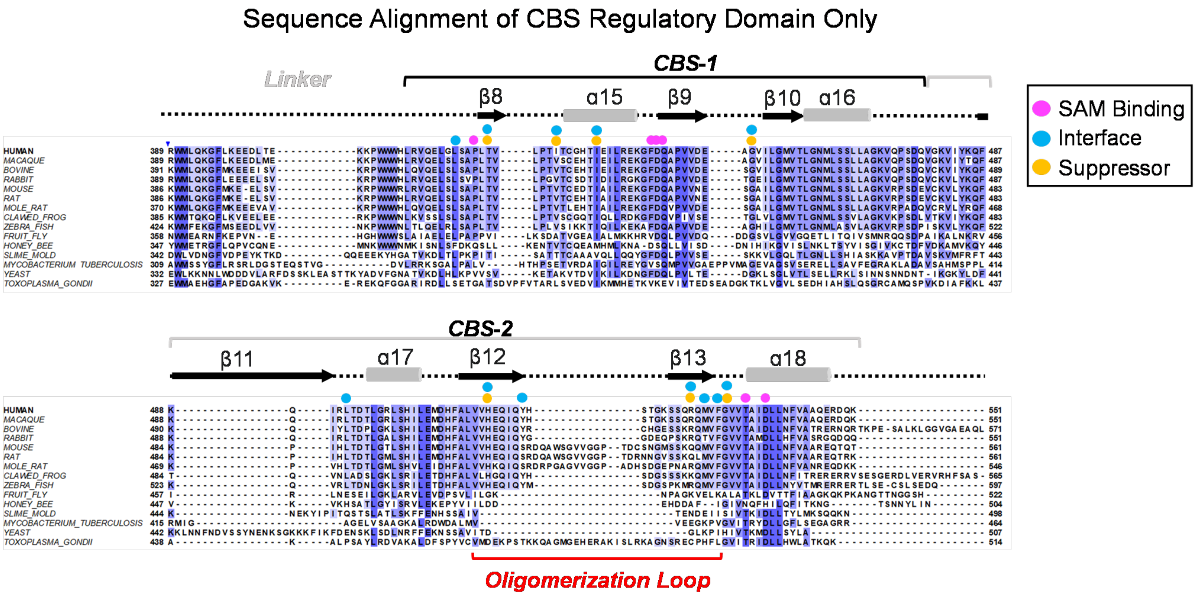

Supplementary Fig. 18 | Sequence alignment of the regulatory domain of CBS enzymes. Sequence alignment of the regulatory domain only of various CBS enzymes. The *CBS-1* and *CBS-2* motifs are indicated.

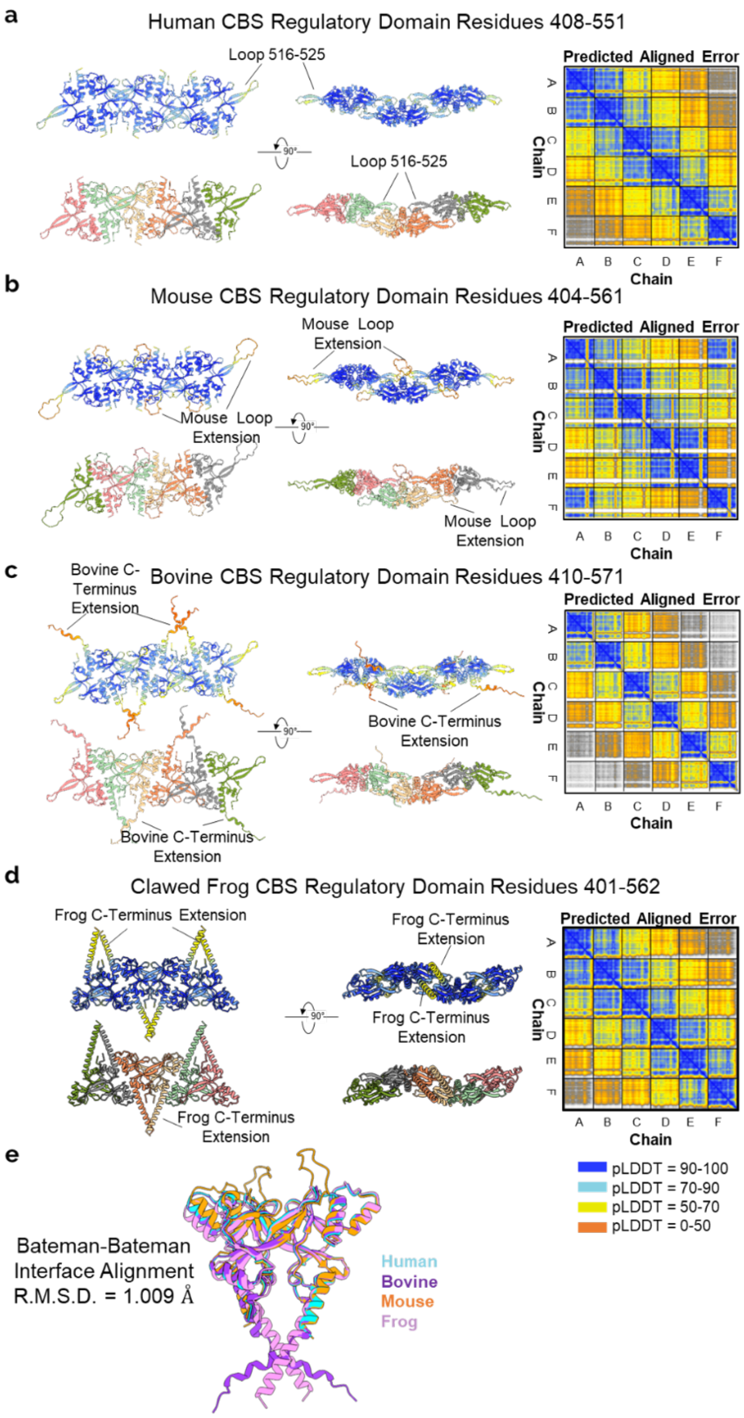

Supplementary Fig. 19 | AlphaFold predicts SAM responsive CBS enzymes polymerise as filaments. a, AlphaFold prediction using six copies of the human CBS regulatory domain (residues 408-551) shows a filament architecture identical to the cryo-EM model of the activated state. **b**, AlphaFold prediction using six copies of the mouse CBS regulatory domain (residues 404-561) shows a filament architecture similar to the cryo-EM model of the human CBS activated state. **c**, AlphaFold prediction using six copies of the bovine CBS regulatory domain (residues 410-571) shows a filament architecture like the cryo-EM model of the human CBS activated state. **d**, AlphaFold prediction using six copies of the African clawed frog CBS regulatory domain (residues 401-562) shows a filament architecture similar to the cryo-EM model of the human CBS activated state. **e**, Structural alignment of the Bateman-Bateman interface from the AlphaFold predicted structures of human, mouse, bovine and frog CBS.

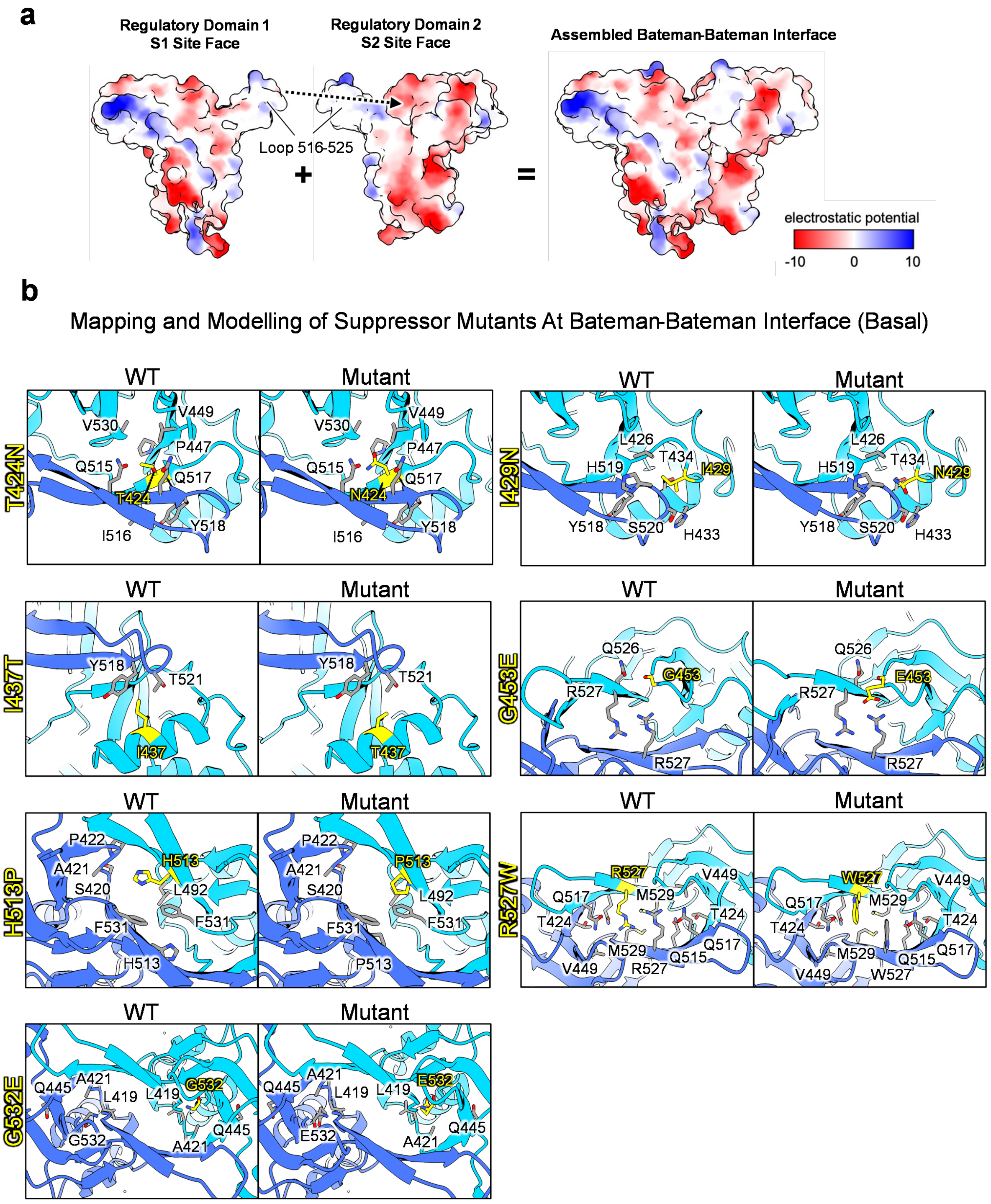

Supplementary Fig. 20 | Hydrophobic interaction of CBS regulatory domains and analysis of suppressor mutations. a, Surface electrostatic potential of the CBS regulatory domain shows that assembly is mainly driven by hydrophobic interactions mediated by the loop 516-525. The interface involves the S2 site face of both regulatory domains **b**, Structural analysis of the seven reported HCU suppressor mutations mapped and statically mutated. All are located at the Bateman-Bateman interface and are predicted to alter oligomerization.

	Helical basal state with His tag (EMDB-19735) (PDB 8S5H)	SPA basal state with His tag (EMDB-19736) (PDB 8S5I)	Helical basal state (EMDB-19737) (PDB 8S5J)	SPA basal state (EMDB-19738) (PDB 8S5K)	Tetramer basal state (EMDB-19739) (PDB 8S5L)
**Data collection and processing**					
Magnification	75,000		150,000		
Voltage (kV)	300		200		
Electron exposure ($e^{-}/Å^2$)	37.85		50.00		
Defocus range (μm)	-0.9 to -3.0		-1.4 to -2.0		
Pixel size (Å)	1.083		0.934		
Symmetry imposed	D1	C2	D1	C2	C2
Initial particle images (no.)	239,739	760,869	749,213	1,190,611	1,190,611
Final particle images (no.)	76,663	113,271	89,761	96,182	67,901
Map resolution (Å)	3.7	3.0	3.9	3.8	3.8
FSC threshold	0.143	0.143	0.143	0.143	0.143
Map resolution range (Å)	3.0-6.0	2.6-6.0	3.3-7.3	3.2-7.1	3.4-7.7
**Refinement**					
Initial model used (PDB code)	4COO	4COO	4COO	4COO	4COO
Model resolution (Å)	4.0	3.5	4.0	3.9	3.9
FSC threshold	0.5	0.5	0.5	0.5	0.5
Map sharpening *B* factor ($Å^2$)	-90	-80	-97	-90	-80
Model composition					
Nonhydrogen atoms	31904	23934	39880	31904	13686
Protein residues	4056	3042	5070	4056	1740
Ligands	8	6	10	8	6
B factors ($Å^2$)					
Protein	76.79	61.93	28.16	58.99	64.75
Ligand	93.49	63.44	39.84	62.78	59.36
R.m.s. deviations					
Bond lengths (Å)	0.003	0.003	0.005	0.003	0.005
Bond angles (°)	0.580	0.529	0.660	0.631	0.659
Validation					
MolProbity score	1.34	1.13	1.88	1.59	1.86
Clashscore	6.20	3.04	10.68	7.15	8.71
Poor rotamers (%)	0.00	0.58	0.14	0.15	0.20
Ramachandran plot					
Favored (%)	98.21	97.84	95.28	96.84	94.21
Allowed (%)	1.59	1.93	4.66	3.04	5.56
Disallowed (%)	0.00	0.23	0.06	0.12	0.23

Supplementary Table 1 | Cryo-EM data collection, refinement, and validation statistics of basal state CBS.

	Global helical activated state (EMDB-19740) (PDB 8S5M)	Focused helical activated state (EMDB-19741)	Focused SPA activated state regulatory domain (EMDB-19742)	Focused SPA activated state catalytic domain
**Data collection and processing**				
Magnification	81,000			
Voltage (kV)	300			
Electron exposure ($e^-/Å^2$)	39.96			
Defocus range (μm)	-0.9 to -3.0			
Pixel size (Å)	1.06			
Symmetry imposed	D1	D1	D1	D1
Initial particle images (no.)	5,240,414	5,240,414	5,240,414	5,240,414
Final particle images (no.)	425,261	425,261	425,261	425,261
Map resolution (Å)	4.0	4.1	4.1	8.3
FSC threshold	0.143	0.143	0.143	0.143
Map resolution range (Å)	3.3-11.0	3.5-13.0	4.0-7.0	6.7-10.3
**Refinement**				
Initial model used (PDB code)	4UUU, 4PCU, AlphaFold			
Model resolution (Å)	6.9			
FSC threshold	0.5			
Model resolution range (Å)	n/a			
Map sharpening *B* factor ($Å^2$)	-126	-126	-169	-200
Model composition				
Nonhydrogen atoms	11630			
Protein residues	1440			
Ligands	10			
B factors ($Å^2$)				
Protein	79.15			
Ligand	73.10			
R.m.s. deviations				
Bond lengths (Å)	0.004			
Bond angles (°)	0.859			
Validation				
MolProbity score	2.39			
Clashscore	31.03			
Poor rotamers (%)	0.56			
Ramachandran plot				
Favored (%)	93.80			
Allowed (%)	6.20			
Disallowed (%)	0.00			

Supplementary Table 2 | Cryo-EM data collection, refinement, and validation statistics of activated state CBS.
