# Supplementary material for: Architecture and regulation of filamentous human cystathionine beta-synthase
Source: Nat Commun. 2024 Apr 4;15:2931. doi: 10.1038/s41467-024-46864-x (PMC10995199; doi:10.1038/s41467-024-46864-x)
Supplement: Supplementary file 2 — Reporting Summary [file 41467_2024_46864_MOESM2_ESM.pdf]

nature portfolio

Corresponding author(s): Thomas J. McCorvie, Wyatt W. Yue

Last updated by author(s): Mar 1, 2024

Reporting Summary

Nature Portfolio wishes to improve the reproducibility of the work that we publish. This form provides structure for consistency and transparency in reporting. For further information on Nature Portfolio policies, see our Editorial Policies and the Editorial Policy Checklist.

Statistics

For all statistical analyses, confirm that the following items are present in the figure legend, table legend, main text, or Methods section.

n/a	Confirmed	
[ ]	[x]	The exact sample size (*n*) for each experimental group/condition, given as a discrete number and unit of measurement
[ ]	[x]	A statement on whether measurements were taken from distinct samples or whether the same sample was measured repeatedly
[ ]	[x]	The statistical test(s) used AND whether they are one- or two-sided<br>*Only common tests should be described solely by name; describe more complex techniques in the Methods section.*
[x]	[ ]	A description of all covariates tested
[x]	[ ]	A description of any assumptions or corrections, such as tests of normality and adjustment for multiple comparisons
[ ]	[x]	A full description of the statistical parameters including central tendency (e.g. means) or other basic estimates (e.g. regression coefficient) AND variation (e.g. standard deviation) or associated estimates of uncertainty (e.g. confidence intervals)
[ ]	[x]	For null hypothesis testing, the test statistic (e.g. *F*, *t*, *r*) with confidence intervals, effect sizes, degrees of freedom and *P* value noted<br>*Give P values as exact values whenever suitable.*
[x]	[ ]	For Bayesian analysis, information on the choice of priors and Markov chain Monte Carlo settings
[x]	[ ]	For hierarchical and complex designs, identification of the appropriate level for tests and full reporting of outcomes
[x]	[ ]	Estimates of effect sizes (e.g. Cohen's *d*, Pearson's *r*), indicating how they were calculated

Our web collection on statistics for biologists contains articles on many of the points above.

Software and code

Policy information about availability of computer code

Data collection	DLS beamline Krios, York Structural Biology Laboratory Glacios, OmegaSTAR MARS Software (BMG Biotech), QuantStudio 3 RT-PCR machine (Thermo Fisher Scientific), MicroCal PEAQ-ITC software v1.30 (Malvern Panalytical), Columbus software v2.4.0
Data analysis	GraphPad Prism, MotionCor2, CTFFIND-4.1, Relion 3.0.8, CryoSPARC-3.1.0, cryoSPARC-3.3.2, COOT, Molrep, UCSF Chimera, PHENIX, Isolde Namdinator, ImageJ, Clustal Omega, AlphaFold2 multimer, ChimeraX

For manuscripts utilizing custom algorithms or software that are central to the research but not yet described in published literature, software must be made available to editors and reviewers. We strongly encourage code deposition in a community repository (e.g. GitHub). See the Nature Portfolio guidelines for submitting code & software for further information.

Data

Policy information about availability of data

All manuscripts must include a data availability statement. This statement should provide the following information, where applicable:

- Accession codes, unique identifiers, or web links for publicly available datasets
- A description of any restrictions on data availability
- For clinical datasets or third party data, please ensure that the statement adheres to our policy

The authors declare that the main data supporting the findings of this study are available within the article and Supplementary Information. Source data are provided with this paper. EM maps and models generated in this study, of CBS basal state (EMD-19735 [https://www.ebi.ac.uk/emdb/EMD-19735], PDB 8S5H [https://www.rcsb.org/structure/8S5H], EMD-19736 [https://www.ebi.ac.uk/emdb/EMD-19736], PDB 8S5I [https://www.rcsb.org/structure/8S5I], EMD-19737

[https://www.ebi.ac.uk/emdb/EMD-19737], PDB 8S5J [https://www.rcsb.org/structure/8S5J], EMD-19738 [https://www.ebi.ac.uk/emdb/EMD-19738], PDB 8S5K [https://www.rcsb.org/structure/8S5K]), degraded CBS tetramer (EMD-19739 [https://www.ebi.ac.uk/emdb/EMD-19739], PDB 8S5L [https://www.rcsb.org/structure/8S5L]) and CBS+SAM activated state (EMD-19740 [https://www.ebi.ac.uk/emdb/EMD-19740], PDB 8S5M [https://www.rcsb.org/structure/8S5M], EMD-19741 [https://www.ebi.ac.uk/emdb/EMD-19741], EMD-19742 [https://www.ebi.ac.uk/emdb/EMD-19742]), have been deposited to the Electron Microscopy Data Bank (EMDB) and Protein Data Bank (PDB).

Research involving human participants, their data, or biological material

Policy information about studies with human participants or human data. See also policy information about sex, gender (identity/presentation), and sexual orientation and race, ethnicity and racism.

Reporting on sex and gender	Not applicable
Reporting on race, ethnicity, or other socially relevant groupings	Not applicable
Population characteristics	Not applicable
Recruitment	Not applicable
Ethics oversight	Not applicable

Note that full information on the approval of the study protocol must also be provided in the manuscript.

Field-specific reporting

Please select the one below that is the best fit for your research. If you are not sure, read the appropriate sections before making your selection.

- [x] Life sciences
- [ ] Behavioural & social sciences
- [ ] Ecological, evolutionary & environmental sciences

For a reference copy of the document with all sections, see nature.com/documents/nr-reporting-summary-flat.pdf

Life sciences study design

All studies must disclose on these points even when the disclosure is negative.

Sample size	The sample size determined by sufficient signal in experiment to ensure confidence in conclusions drawn from data. For functional assays, sample sizes for each experiment are shown as individual points. For Cryo-EM structural determination the number of micrographs collected for each data set is provided in Supplementary Information. For cell imaging, the number of cells evaluated is provided in Figure 5 legend.
Data exclusions	No data excluded.
Replication	Activity and DSF measurements were carried out in technical replicates of n = 4 or 6 as stated in figure legends. ITC were carried out in technical replicates n=2.
Randomization	Not applicable - no experimental groups were involved.
Blinding	Not applicable - no group allocation was involved.

Reporting for specific materials, systems and methods

We require information from authors about some types of materials, experimental systems and methods used in many studies. Here, indicate whether each material, system or method listed is relevant to your study. If you are not sure if a list item applies to your research, read the appropriate section before selecting a response.

Materials & experimental systems

n/a	Involved in the study	
[x]	[ ]	Antibodies
[ ]	[x]	Eukaryotic cell lines
[x]	[ ]	Palaeontology and archaeology
[x]	[ ]	Animals and other organisms
[x]	[ ]	Clinical data
[x]	[ ]	Dual use research of concern
[x]	[ ]	Plants

Methods

n/a	Involved in the study	
[x]	[ ]	ChIP-seq
[x]	[ ]	Flow cytometry
[x]	[ ]	MRI-based neuroimaging

Eukaryotic cell lines

Policy information about cell lines and Sex and Gender in Research

Cell line source(s)	The PC-3 cell lines were obtained from Brazilian Biosciences National Laboratory through ATCC. Cell lines BT549, MCF7, MDA-MB-231, and hFB were acquired from ATCC. MEF cells were donated by Dr. Angela Saito.
Authentication	They have been verified and are not listed among the known misidentified cell lines according to the International Cell Line Authentication Committee (ICLAC).
Mycoplasma contamination	All cell lines neagative for mycoplasmia contamination
Commonly misidentified lines (See ICLAC register)	none

Plants

Seed stocks	n/a
Novel plant genotypes	n/a
Authentication	n/a
