# Supplementary material for: Architecture and regulation of filamentous human cystathionine beta-synthase
Source: Nat Commun. 2024 Apr 4;15:2931. doi: 10.1038/s41467-024-46864-x (PMC10995199; doi:10.1038/s41467-024-46864-x)
Supplement: Supplementary file 6 — Description of Additional Supplementary Files [file 41467_2024_46864_MOESM6_ESM.pdf]

File Name: SupplementaryMovie1.mp4

Description: Morph of the Bateman-Bateman interface from the basal and SAM bound activated structure of CBS. The two neighbouring Bateman domains are represented as cartoons. SAM is represented as balls and sticks and is coloured pink.

File Name: SupplementaryMovie2.mp4

Description: Morph of one full turn of the basal and SAM bound activated structures of CBS. One CBS dimer is coloured light and dark blue. Neighbouring CBS dimers are coloured grey.
